# Environmentally Sensitive Fluorescent Sensors Based on Synthetic Peptides

**DOI:** 10.3390/s100403126

**Published:** 2010-03-31

**Authors:** Laurence Choulier, Karin Enander

**Affiliations:** 1 Biosensor group, CNRS / Université de Strasbourg, IREBS, Parc d’Innovation, Boulevard Sébastien Brandt, BP 10413, 67412 Illkirch cedex, France; 2 Division of Molecular Physics, Department of Physics, Chemistry and Biology, Linköping University, 581 83 Linköping, Sweden; E-Mail: karen@ifm.liu.se

**Keywords:** peptide biosensors, environmentally-sensitive fluorophore

## Abstract

Biosensors allow the direct detection of molecular analytes, by associating a biological receptor with a transducer able to convert the analyte-receptor recognition event into a measurable signal. We review recent work aimed at developing synthetic fluorescent molecular sensors for a variety of analytes, based on peptidic receptors labeled with environmentally sensitive fluorophores. Fluorescent indicators based on synthetic peptides are highly interesting alternatives to protein-based sensors, since they can be synthesized chemically, are stable, and can be easily modified in a site-specific manner for fluorophore coupling and for immobilization on solid supports.

## 1. Introduction

A molecular biosensor [1] is a molecule composed of a biological recognition element (receptor) covalently associated with a transducer, generally a fluorophore, for signaling. The receptor is selected to specifically recognize a molecule of interest (analyte), while the fluorophore responds to the recognition event and transforms it into a measurable signal. The molecular biosensor can be distinguished from a chemosensor by the nature of the receptor, which is typically a biomacromolecule such as a nucleic acid (DNA or RNA), or a protein (often an enzyme or an antibody). Fluorescent molecular biosensors are mainly designed for the detection and quantification of analyte, with potential applications in the fields of medicine, agro-industry, defense or protection of the environment, but are also exploited to understand biomolecular events.

**Figure 1.**
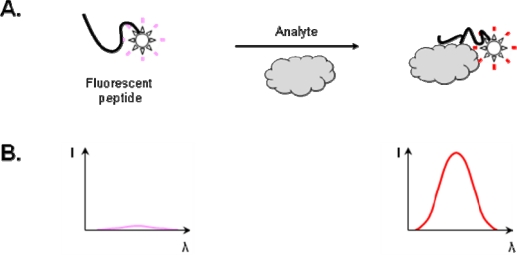
Principle of a fluorescent peptide biosensor. **A)** Schematic drawing showing the effect of analyte binding. The fluorophore attached close to the binding site responds to a microenvironmental change. **B)** Binding of analyte is detected by changes in the fluorescence emission spectrum. Adapted from [18], with permission from the American Chemical Society.

Compared to an immunoassay, which requires indirect labeling and multistep reactions, the detection of an analyte by a fluorescent biosensor is simple, direct and can be appreciated in real-time. The principle of sensing is illustrated in Figure 1. When an environmentally sensitive fluorophore is coupled near the binding site of a receptor, it will respond to changes in the physico-chemical environment induced by complex formation, by altering its fluorescence properties. Typically, polarity variations in the immediate vicinity of the fluorophore result in quenching or enhancement of the fluorescence and/or a wavelength shift in the emission spectrum. Recent reviews describe the structures, key physical parameters (extinction coefficients, excitation and emission wavelengths, quantum yields, size, hydrophobicity, stability) and advantages of some of the most common environmentally-sensitive fluorophores [2], or the design of biosensor molecules based on proteins [3]. While implemented with many different types of protein receptors, analytes and fluorophores, site-specific incorporation of fluorophores in proteins remains a challenge. The most widely used approach to covalently couple an extrinsic fluorophore to a recombinant protein involves the replacement of a less reactive amino acid residue with cysteine, followed by dye coupling to the free thiol group of the purified protein in buffer. This approach is straightforward, but difficult to implement on a general basis because mutations and coupling can lead to poor expression levels, deleterious effects on analyte binding, poor stability, aggregation or unfolding. Moreover, side reactions between thiol-reactive fluorophores and lysine side chains have been observed [4,5]. Other approaches to coupling a fluorophore at a specific site of proteins involve nonsense suppression [6], active-site-selective labeling [7–9], post-photoaffinity labeling modification [10–12], and, for chemically solid-phase synthesized proteins, the Suzuki-coupling reaction [13] and native-chemical ligation of peptides [14].

Protein-based environmentally-sensitive fluorescent biosensors have been successfully used for analyte detection and quantification with affinities varying from 0.2 nM to 150 mM, sometimes even in immobilized formats [14–17]. Still, synthetic peptides are interesting alternatives as receptors, because they provide obvious advantages over proteins in terms of production and stability. Peptide synthesis is straightforward and cost-effective and peptide-dye constructs can be manufactured at large scale. Moreover, synthetic peptides are robust, offer an even larger chemical versatility than recombinant proteins and are easily modified in a site-specific manner by means of orthogonal protection group strategies. Thus, peptides should have a great potential as components in molecular biosensors.

This review will specifically focus on biosensor constructs based on synthetic peptides and designed for the detection of biomolecular analytes by transduction *via* environmentally sensitive fluorescent dyes. Detection based on energy transfer (FRET) or changes in anisotropy will not be considered. The peptide may either serve as the recognition unit to provide ‘intrinsic recognition’ (Section 2) or as a scaffold onto which other recognition elements can be grafted (‘extrinsic recognition’, Section 3).

## 2. Synthetic Peptide Sensors: Peptides as Recognition Elements

Half of the reported protein biosensors are based on bacterial periplasmic binding proteins and target sugars, the reason being that these proteins are well described and that sugar binding induces a pronounced conformational change that may result in local changes in the chemical environment in many parts of the protein. In contrast, most of the singly-labeled fluorescent peptide biosensors target proteic or peptidic analytes. Binding-related peptide sensors constructed by labeling of an amino acid residue and by incorporation of a fluorescent amino acid are listed in Tables 1 and 2, respectively, together with targeted analytes, sensor affinities and performances. Sensors for enzymes, where the fluorescence signal is changed in response to the enzymatic activity (discussed in Section 2.1.), are not included.

This section will describe how and for what purpose peptide biosensors are constructed.

### 2.1. Applications of Peptide Biosensors

Protein chips are expected to become important tools for direct analyses of biomolecular function and interaction in a high-throughput fashion. Hisakazu Mihara and his team contribute to this development by designing protein-detection systems, where peptide-based biosensor molecules with defined secondary structures are used as capture agents. Interactions are reported by environmentally sensitive fluorophores attached to the peptides. Libraries of peptides with β-strand (16 peptides, [25]), β-loop (126 peptides, [24]) and α-helix (20 peptides, [27]) structures were synthesized. In proteins, the solvent accessible part of these secondary structure elements is often implicated in the recognition of protein partners. Peptides were introduced into separate wells of a microplate, either involving covalent immobilization *via* an N-terminal cysteine [24,25] or adsorbed [27]. The resulting peptide arrays were used to produce characteristic protein fingerprints (PFP), allowing discrimination between a range of proteins. The proteins could be detected down to 1.2 fmol [27]. Tomizaki and Mihara [26] developed a fluorescence sensing system for the detection of proteins using a photochromism-based assay (P-CHROBA) technique. Spiropyran derivatives were attached to the N-termini of eight peptides. When irradiated by UV or visible light, spiropyran undergoes a reversible transition to a highly fluorescent merocyanine form with rate constants that depend on the microenvironment of the dye. Changes in these constants were used to detect complex formation between the spiropyran-containing peptides and six different proteins. Even this small number of peptides gave rise to unique PFPs and allowed for successful discrimination between the proteins.

Many peptide-based biosensors were constructed to gain insights into peptide-protein interaction systems. Fluorophore-peptide conjugates were used as probes to resolve the interaction kinetics of peptide-chaperone (*E. coli* DnaK and SecB) interactions and to gain insights into the relative polarity of the peptide binding site [19,20]. Similarly, Wearsch *et al.* [28] investigated complex formation between the chaperone GRP94 and a peptide derived from the vesicular stomatitis virus labeled with an environmentally sensitive fluorophore. By using peptides derived from cholecystokinin (CCK) and modified with the fluorophore Alexa, Laurence J Miller’s group demonstrated that the environment of the fluorophore is different when the peptide is bound to type A [21] and type B [22] CCK receptors. By incorporating the fluorescent amino acid DANA (6-(2-dimethylaminonaphthoyl)alanine) in the N-terminus, the mid-region, or the C-terminus of the peptide, pronounced differences in the mechanism of CCK binding and activation of these structurally related receptors were shown [34]. Further, Harikumar *et al.* [23] examined the binding environment of a peptide agonist to family B of G protein-coupled secretin receptors by attaching Alexa Fluor 488 at four different positions throughout the pharmacophore. Peptide-based biosensors were also used to probe the phosphorylation-dependent binding of an octapeptide to the 14-3-3 protein (see Section 2.2.1) and to study intramolecular interactions [32].

**Scheme 1.**
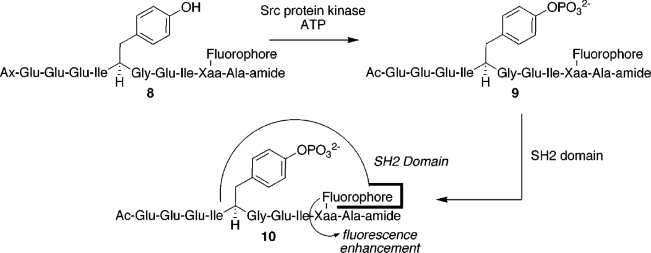
Fluorescent sensing of Src protein kinase based on binding of an SH2 domain to the sensor peptide containing the kinase recognition sequence and a target tyrosine. The affinity of the SH2 domain for the peptide is increased upon phosphorylation, and SH2 binding induces a change in the chemical environment surrounding the fluorophore. From [41].

Several peptide biosensors equipped with environmentally-sensitive fluorophores were designed for the detection of enzymes and for monitoring their activity. David S. Lawrence and colleagues designed functionalized peptide libraries for the identification of efficient biosensor systems for protein kinases. Sensing of Src kinase was based on phosphorylation-driven disruption of stacking interactions between an extrinsic fluorophore and the target tyrosine residue in a peptide substrate [39,40]. In a different design, an environmentally sensitive fluorophore was covalently attached to a tyrosine-containing peptide substrate recognized by Src kinase and the assay was performed in the presence of the phosphotyrosine binding domain Lck-SH2 [41]. Upon tyrosine phosphorylation, the fluorophore experienced changes in the local environment caused by the peptide binding to Lck-SH2 (Scheme 1). A similar approach was used for the detection of serine phosphorylation by the cAMP-dependent protein kinase [42]. Barbara Imperiali and colleagues designed a generic kinase sensor containing a variable enzyme recognition sequence and a fluorescent amino acid residue, Sox (from 8-hydroxy-5-(N,N-dimethylsulfonamido)-2-methylquinoline), which displays chelation-enhanced emission when binding Mg^2+^. Phosphorylation of a target serine, threonine or tyrosine residue increased the affinity of Sox for the metal ion, which resulted in enhanced fluorescence intensity [43,44].

Fluorescent peptide reporters for histone acetyltransferase were designed by synthesizing a 20-residue peptide corresponding to the N-terminal part of histone H4 followed by functionalization with dansyl or fluorescein [45]. Charge reduction of the peptide following acetylation of target lysines resulted in a small increase in the emission from the fluorophores. Along similar lines, biosensor peptides for arginine methyltransferase 1 were also designed [46].

### 2.2. Construction of Peptide Biosensors

#### 2.2.1. Strategies for the incorporation of fluorescent dye in the peptide

Two main strategies for covalent incorporation of the fluorophore in the peptide can be distinguished. The first one involves post-synthetic coupling at the N-terminal amine [21–23,28,30] or at the side chains of cysteines [19,29] or lysines [24–27,31].

**Scheme 2.**
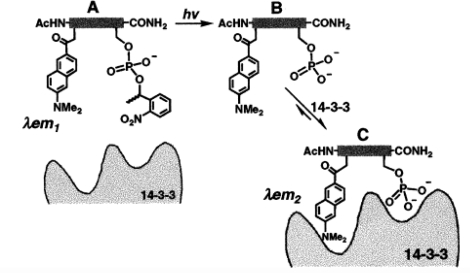
Probing of the phosphorylation-dependent binding of an octapeptide containing the fluorescent amino acid DANA to a 14-3-3 protein. **A)** The caged phosphopeptide is unable to bind 14-3-3. The maximum emission wavelength (λ_em1_) of DANA is 522 nm. **B)** Irradiation of the caged phosphopeptide releases free phosphoserine-containing peptide. **C)** Released phosphoserine-peptide binds to the protein, thereby modulating the fluorescence properties of DANA (λ_em2_ = 501 nm). From [33].

The second strategy involves the incorporation of unnatural fluorescent amino acids during peptide synthesis. Barbara Imperiali’s group has developed the functionalized amino acids DANA [33] and 4-DAPA (4-N,N-dimethylaminophtalimidoalanine, [35]), and used them to probe the phosphorylation-dependent binding of an octapeptide to a 14-3-3 protein. The 14-3-3 proteins are essential intermediates in cell cycle regulation, acting through phosphorylation-dependent protein-protein interactions. A caged fluorescent octo-phospho-peptide was synthesized that exposed a phosphoserine residue upon irradiation, allowing the peptide to bind to the 14-3-3 protein and modulate the emission properties of the fluorescent amino acid (Scheme 2). A shift in the maximum emission wavelength was observed, along with a 4- and 6-fold increase in the emission intensity for the DANA- and 4-DAPA-containing peptides, respectively.

**Figure 2.**
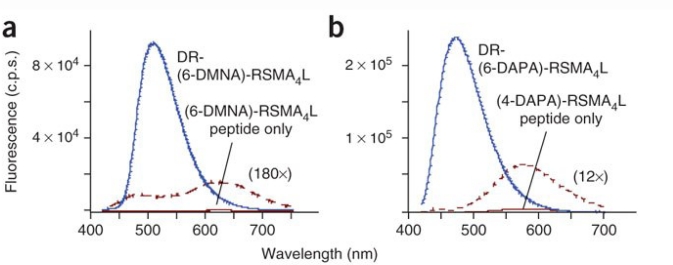
Fluorescence emission spectra of a) a (6-DMNA)-peptide and b) a (4-DAPA)-peptide (**b**) and their complexes with a class II MHC protein (DR) are shown. Free peptide spectra are shown also on an expanded scale. Note the shift in emission wavelength λ_max_ upon binding of the peptide to protein, as well as the increase in the emission intensity. From [38], reprinted by permission from Macmillan Publishers Ltd (licence number 2341811044647).

The amino acid 6-DMNA (6-N,N-dimethylamino-2,3-naphtalimidoalanine) was introduced adjacent to the conserved binding determinants of several peptides and exhibited up to an 11-fold change in fluorescence emission intensity upon binding to selected Src homology 2 (SH2) phosphotyrosine binding domains [37]. Much larger signal enhancements were observed for the binding of peptides containing 4-DAPA (470-fold) and 6-DMNA (1100-fold) to class II MHC proteins [38] (Figure 2). In two other papers, DANA was introduced in place of a phenylalanine to construct a fluorescent δ-opioid antagonist [32] and at three positions of a CCK-binding peptide to provide insights into distinct modes of binding and activation of type A and B CCK receptors [34].

#### 2.2.2. Position of the fluorophore in the amino acid sequence

It is difficult to predict the optimal position for labeling. It should be chosen so that the microenvironment of the fluorophore is altered upon analyte binding. In protein biosensors, this is achieved either (i) by analyte-dye contacts in or (ii) near the analyte binding site, or (iii) by conformational effects occurring over substantially longer intra-protein distances, affecting a fluorophore located distantly from the analyte binding site [47,48]. The design of a molecular sensor thus requires some knowledge about the interaction, gained from structural or functional studies, in order to identify positions for which fluorescent labeling allows analyte detection without perturbing its binding. Not only the position, but also the nature of the dye plays a critical role in the performance of a biosensor. It has been observed that simply changing the fluorophore at the same site of a protein can lead to differences in binding parameters [8,18,49,50]. A combinatorial approach with various fluorophores is recommended to optimize sensitivity [51] and affinity.

Site-dependent differences in fluorescence response upon analyte binding to a peptide biosensor were quantified in three different studies.

**Figure 3.**
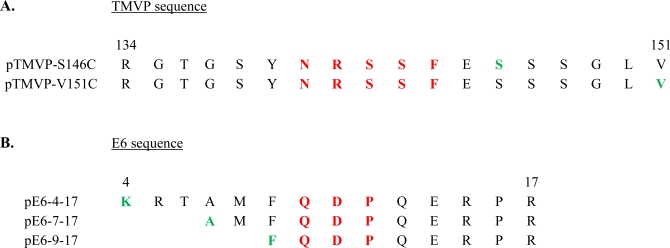
Sequences of the peptides corresponding to A) amino acids 134–151 of the TMV protein [29], and **B)** amino-acids 4–17, 7–17 and 9–17 of oncoprotein E6 [30]. Residues most important for analyte binding are shown in red, while positions targeted for fluorophore labeling are shown in green. Post-synthetic coupling of the fluorophore was performed **A)** at the side chains of cysteines replacing residues S146 and V151, and **B)** at the N-terminal amine.

Enander *et al.* [29] constructed a peptide-based ratiometric biosensor for the detection of an antibody fragment. Based on a detailed characterization of the functional epitope corresponding to residues 134–151 of the tobacco mosaic virus protein (TMVP), two positions were targeted (Figure 3A) for labeling with a two-band fluorophore, one in the immediate vicinity of positions mapped as essential for binding (position 146) and one in the C-terminus (position 151). Only one out of these two constructs behaved as a biosensor. When the peptide was labeled in the C-terminus, seven amino acids away from the essential residues of the epitope, the intensity ratio of the two emission bands changed by 40% upon analyte binding, while labeling two amino acids away from the residues most important for binding resulted in a construct that completely lacked ratiometric sensing ability. Choulier *et al.* [30] performed a similar study on a different antigen-antibody system. Three peptides, corresponding to the N-terminal sequence of the oncoproteine E6 of human papillomavirus (HPV) 16, were labeled at different distances from the epitope (Figure 3B): zero, two and five residues away from the first position in the sequence mapped as important for binding of the antibody fragment. One of these three peptidic constructs possessed sensing properties, namely the peptide for which the dye was coupled two residues away from the epitope. Addition of the antibody fragment to this peptide caused a 47 % decrease in the ratio of its two emission bands.

These two examples indicate that the fluorescence response largely depends on the site of fluorophore incorporation, and also that its distance to residues essential for analyte binding cannot be used as a simple parameter for sensor optimization. Similar results have been presented for protein biosensors earlier [48,50,52–58].

Recently, Sainlos *et al.* [36] developed a general screening strategy in the design of environmentally sensitive peptidic probes for PDZ domains. The design strategy includes two steps: (1) development of a peptide library to screen for the optimal site for fluorophore attachment and (2) screening of different peptide sequences for improved affinity and specificity while keeping a fixed fluorophore position. The initial library, consisting of 11 decamer peptides derived from the C-terminal sequence of stargazin (^−9^NTANRRTTPV^0^) that included 4-DAPA derivatives in eight different positions, was screened against three PDZ constructs. Positions 0 and −2 were not tested as they are essential for binding. Further optimization was carried out by adjusting the length of the linker bearing the dye (from one to three methylene groups). After this first step, out of eight coupling locations, three produced sensors with a <10-fold increase in fluorescence, two with a 10–20-fold increase and three with a >25-fold increase. Maximum fluorescence increase was obtained with the fluorescent amino acid in position −5, bearing a 2-methylene linker (β). In a second step, this position and linker size were kept fixed in the C-terminal sequences of a series of four known PDZ binders. The highest fluorescence increase was 265-fold for the GluR1 sequence (^−9^SGδPβGATGV^0^, δ being norleucine) with the PDZ domain Shank3. Both the nature of the fluorophore and its site of conjugation seem important for maximal signal change.

## 3. Synthetic Peptide Sensors: Recognition Element Grafted on a Peptide Scaffold

In the examples given in Section 2, the sequence of the peptide was chosen in order to provide the recognition site for the analyte. A different role for the peptide has been presented in a series of papers by the groups of Akihiko Ueno and Lars Baltzer. Here, the folding propensity of polypeptides is a key property that makes them useful as scaffolds, onto which non-peptidic molecules for recognition can be grafted along with fluorophores for signaling. A well-defined structure provides directionality of incorporated groups, which is an important parameter in the design and optimization of biosensor performance. Also, the distance between recognition unit and fluorophore can be systematically varied by addressing different amino acid side chains in the scaffold.

**Figure 4.**
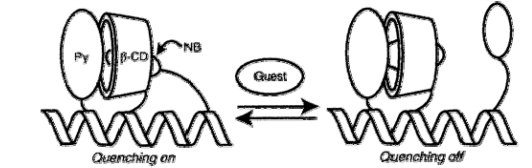
Schematic representation of a scaffold-based biosensor with β-cyclodextrin (β-CD) acting as the recognition element. Upon binding of the analyte (guest), the quencher nitrobenzene (NB) is displaced from the β-CD binding site and the emission from the fluorophore (pyrene, Py) is enhanced. From [63], with permission from the American Chemical Society.

A number of biosensor molecules based on a 17-residue, *de novo* designed α-helical peptide were presented, where different cyclodextrins were employed for recognition of bile acids and aliphatic alcohols [59–67]. The sensing principle was based on the disruption of an inclusion complex between cyclodextrin and a fluorophore upon binding of the analyte (guest). In the simplest design, dansyl or dimethylaminobenzoyl was attached in position i, i + 4 or i, i + 7 with respect to a β-cyclodextrin moiety, which corresponds to the two groups being separated by one or two turns of the helix scaffold while oriented similarly in space [59,65]. This arrangement allowed the fluorophore to interact with the hydrophobic interior of the cyclodextrin moiety. Upon introduction of a guest molecule, e.g., ursodeoxycholic acid, displacement of the fluorophore was reflected in quenching of its emission. In a different construct, α- or β-cyclodextrin was coupled to the scaffold along with pyrene and its quencher nitrobenzene [63,66]. The signaling principle was based on nitrobenzene and the analyte competing for the binding site inside cyclodextrin, affecting the distance between fluorophore and quencher and thus the fluorescence intensity (Figure 4). Yet another design approach involved functionalization with two molecules of naphthalene or pyrene, and analyte binding was monitored from excimer fluorescence [60–62,64]. Bile acids and ring-structured alcohols were sensed using constructs with β- [59,63–65,67] or γ-cyclodextrin [60–62] while α-cyclodextrin was used for short chain aliphatic alcohols [66].

In a similar approach, *de novo* designed helix-loop-helix polypeptides that dimerize into four-helix bundles upon folding were functionalized to become molecular biosensors for protein analytes. In the initial design, dansyl and a benzenesulfonamide derivative were attached to separate helices of the scaffold [68,69]. The presence of carbonic anhydrase, which binds specifically to benzenesulfonamide, was accompanied by a disruption of the dimer [70] and a significant intensity increase of the dansyl fluorescence.

## 4. Affinity Characterization of the Interaction between Biosensor and Analyte

The equilibrium dissociation constant (K_D_) of interactions between peptide biosensors and their analytes can be determined from the hyperbolic binding curves obtained by titration of a constant concentration of peptide biosensor with increased concentrations of analyte [13,19,20,27,31,35]. Affinities determined in this way vary from low nM to 500 μM depending on the system under study. In two studies [30,36], competition binding assays with non-fluorescent peptides recognizing the same analyte as the peptide biosensor further complemented titration experiments and allowed for determination of the relative affinities of unlabeled peptides for their protein analytes. The binding affinities were also independently evaluated by using non-fluorescence-based methods; isothermal titration calorimetry (ITC, [36]) and surface plasmon resonance (SPR, [30]). In both cases, K_D_ values determined by ITC or SPR were in the same range as those determined by fluorescence-based procedures.

Based on the helix-loop-helix scaffolds designed by Baltzer and colleagues, biosensors with affinities towards carbonic anhydrase spanning three orders of magnitude were designed by varying the length of an aliphatic spacer attached to the benzenesulfonamide moiety [69,71]. Although interactions between the spacer and a hydrophobic pocket in the protein contributed to binding, the scaffold itself played an important role in providing both sterical constraints and chemical functionalities that affected the affinity [71].

## 5. Limit of Detection

Singly labeled reagentless biosensors based on proteins and used for analyte quantification displayed limits of detection (LOD) in the submillimolar range for maltose [54], and in the submicromolar range for glucose [72], glutamine [17,73], and β-lactam antibiotic [74].

The LOD of analyte by peptide biosensors was reported only in a few cases. The protein detection method established with folded peptides (see Section2.1) allowed for the detection of 5 μg/mL (# 90 nM) of α-amylase [24]. The LOD of the antibody fragment scFv1F4_Q34S_ by the pE6 peptide biosensor [30] was 15 nM in buffer, and 50–100 nM in a more complex medium (bovine serum albumin (BSA) 100 μg/mL). Normal concentrations of specific antibodies and BSA in serum are around 50 μg/ml (330 nM) and 35–50 mg/ml, respectively.

## 6. Perspectives for Peptide Biosensors

The performances of peptide and protein sensors were recently compared [30] based on various antigen-antibody interactions that display affinities in the 0.1–20 nM range. In these studies, the receptor was either an antibody [52,53], or a peptide antigen [29,30], but the analytes were large in all cases (between 14 and 54 kDa). A biosensor was defined as operational if it showed a >35% fluorescence intensity variation upon analyte binding. Out of the total number of sensors constructed in each study (maximum 10 for antibody receptors and 3 for peptide receptors), 20–50% behaved as biosensors. In these studies, the proportion of efficient fluorescent sensors was therefore in the same range whether the receptor was a protein or a peptide. This observation makes peptide biosensors promising tools for developing new applications. With the availability of automatedchemical synthesis of long peptide fragments at high scale (100 mg of a 100 amino acid peptide can be routinely synthesized at >99% purity) and low price, rational or combinatorial peptide libraries can be straightforwardly produced for the optimization of recognition sites and positions for fluorophore attachment. However, the main challenge in the future will be to make peptide-based fluorescent biosensors useful for sensitive detection of bio-analytes in complex media (e.g., serum). In order to accomplish this, problems with interference from non-analyte components in the sample have to be addressed. Specifically, the possibility of enzymatic peptide degradation and of non-specific binding of sample components to the peptide, causing irrelevant sensor signals, has to be taken into consideration. The frequent problem of autofluorescence can be avoided by employing long-wavelength dyes, which are usually very bright although they may be less sensitive to environmental changes compared to fluorophores excited in the UV region. Ratiometric sensor peptides, where the relative rather than the absolute intensity change upon analyte binding is monitored, should also be very useful in this respect.

In conclusion, fluorescent sensor peptides have already proven useful in a number of applications, ranging from analyte detection to elucidation of molecular details of protein-peptide and protein-protein interactions. It is expected that their general applicability with respect to analyte quantification – beyond proof-of-concept – will be expanded by combining combinatorial methods for peptide design with further improvement of fluorophores and fluorescent amino acids in terms of sensitivity to environmental changes. The most robust protein detection system will then probably rely on fingerprints obtained from arrayed sensor peptides.

## Figures and Tables

**Table 1.** Environmentally sensitive fluorescent peptide biosensors constructed by labeling of an amino acid.

**System characteristics**			**Sensor affinity and performance**		**References**
**Analytes**	**Receptors**	**Fluorophores**	**Maximum fluorescent signal change upon analyte-receptor interaction**	***K*_D_ of the corresponding analyte-receptor complex**	
DNaK chaperone	Targeting sequence of the precursor of mitochondrial aspartate aminotransferase	Acrylodan	4-fold	1.4 μM	[19]
SecB chaperone	Bovine pancreatic trypsin inhibitor	Acrylodan	3.4 -fold	5.4 nM	[20]
Cholecystokinin (CCK) receptor	Peptides agonist and antagonist of the CCK receptor	Alexa, NBD^a^, Acrylodan	NR	ND^d^	[21]
Cholecystokinin (CCK) receptor	Peptides agonist of the CCK receptor	Alexa^488^	NR	ND^d^	[22]
Secretin receptor	Analogues of the hormone secretin	Alexa^488^	NR	ND^d^	[23]
α-amylase^#^	Library of designed loop peptides	Fluorescein	> 4-fold	1.1 μM	[24]
β-lactoglobulin^#^	Mini-library of designed β-strand peptides	Fluorescein	2.5-fold	ND	[25]
PKA, α-amylase, β-galactosidase, lysozyme, hexokinase, S-100	Peptides derived from substrates of 4 kinases (PKA, c-Src kinase, c-Abl tyrosine kinase & PKC)	Spiropyran	NR	ND	[26]
Calmodulin^#^	Mini-library of designed α-helical peptides	TAMRA^b^	4-fold	1.5 μM	[27]
GRP94	VSV8, the immonudominant peptide epitope of the vesicular stomatitis virus	Acrylodan, Nile-red	NR	ND^d^	[28]
Fab 57P	Sequence 134–151 of the tobacco mosaic virus coat protein	3-hydroxychromone^c^*	1.4-fold^e^	2.4 nM	[29]
scFv 1F4	N-terminus sequence of the E6 protein of human papillomavirus 16	3-hydroxychromone^c^*	1.5 -fold^e^	1nM	[30]
Double-stranded DNA	Polypeptide derived from the Hin recombinase of *Salmonella typhimurium*	Oxazole yellow	>1.1-fold	10 nM	[31]

NR. Not reported; ND. Not determined;

a 4-nitrobenzoxadiazole;

b 5-(and-6)-carboxytetramethylrhodamine;

c 2-(2-furanyl)-3-hydroxychromone

d *K*_i_ or EC50 values were reported;

e This number refers to changes in the ratio of the intensities of the twoemission bands characteristic of this dye;

# Main protein used as analyte;

* Ratiometric fluorophores are inherently quantitative as the ratiometric signal is independent of probe concentrations.

**Table 2.** Environmentally sensitive peptide biosensors constructed by incorporating a fluorescent amino acid in the peptide sequence.

**System characteristics**			**Sensor affinity and performance**		**References**
**Analytes**	**Receptors**	**Fluorescent amino acids**	**Maximum fluorescent signal change upon analyte-receptor interaction**	***K*_D_ of the corresponding analyte-receptor complex**	
δ-opioid receptor	δ-opioid antagonists	DANA^a^ (Aladan)	NR	ND^d^	[32]
14-3-3 protein	Caged phosphopeptides	DANA^a^ (Aladan)	4-fold	700 nM	[33]
Cholecystokinin (CCK) receptor	Peptides agonist of the CCK receptor	DANA^a^ (Aladan)	NR	ND^d^	[34]
14-3-3 protein	Phosphopeptides	4-DAPA^b^	6-fold	4.6 μM	[35]
PDZ domains	C-terminal sequence of stargazin, CRIPT, NR2a and GluR1	4-DAPA^b^	265-fold	0.2 μM	[36]
SH2 phosphotyrosine-binding domains	SH2 domains	6-DMNA^c^*	11-fold	2.4 μM	[37]
Class II MHC proteins	HLA-DR-binding peptides	4-DAPA^b^ and 6-DMNA^c^*	1100-fold	ND^d^	[38]

NR. Not reported; ND. Not determined;

a 6-(2-dimethylaminonaphthoyl)alanine;

b 4-N,N-dimethylaminophtalimidoalanine;

c 6-N,N-dimethylamino-2,3-naphtalimidoalanine;

d *K*_i_ or EC50 values were reported;

* Can be used as ratiometric fluorophore
